# A Simulation-Based Program for Preparedness for COVID-19 at a Pediatric Tertiary Hospital in Saudi Arabia

**DOI:** 10.7759/cureus.13131

**Published:** 2021-02-04

**Authors:** Tarek R Hazwani, Zahra Al Hassan, Ahlam Al Zahrani, Ayham Al Badawi

**Affiliations:** 1 Medical Research, King Abdullah International Medical Research Center, Riyadh, SAU; 2 Pediatric Critical Care, King Abdulaziz Medical City, Ministry of National Guard - Health Affairs, Riyadh, SAU; 3 College of Medicine, King Saud Bin Abdulaziz University for Health Sciences, Riyadh, SAU; 4 Nursing, Ministry of National Guard - Health Affairs, Riyadh, SAU; 5 Infection Prevention and Control, Ministry of National Guard - Health Affairs, Riyadh, SAU

**Keywords:** simulation, covid-19, preparedness, health care, crisis

## Abstract

Background

COVID-19 has created major challenges for academic education and clinical training, as well as on routine, emergency, and elective patients who have been impacted by the health service’s response to COVID-19. Simulation helps recognize and correct both active and latent threats in health institutions.

Objectives

This study aimed to describe the implementation steps, challenges, and solutions for simulations to make a difference in hospital operational readiness in the response to COVID-19.

Methods

We conducted a series of in situ simulations in different areas of the hospital to deal with COVID-19 patients. We designed seven scenarios to include different clinical situations of pediatric COVID-19 cases, such as emergency room triage and respiratory support.

Results

In total, seven simulation-based drills were conducted during March 1-30, 2020, and 89 healthcare professionals participated in these sessions. Many of the revealed latent threats were regarding teamwork, workflows, and adherence to infection control measures.

Conclusion

We found that in situ simulations helped to identify multiple latent hazard issues. Simulations have a great positive impact on hospital preparedness for the COVID-19 crisis in the pediatric field. Video-recorded simulations method is a good alternative to maintain medical supplies during the COVID-19 crisis.

## Introduction

The COVID-19 pandemic has created many challenges that have necessitated changes in working practices among the entire world population, and particularly in healthcare settings.

This pandemic has had a detrimental effect on routine, emergency, and elective patients who have been impacted by the health service’s response to COVID-19. Further, patient and staff safety is essential in any health institution during this crisis, and the dynamic and quick progression of the COVID-19 pandemic requires a highly adaptable and resilient healthcare system to manage the rapid impact ahead before a lot of COVID-19 patients seek treatment [1].

Simulation helps recognize and correct both active and latent threats in the local environment and health institutions [2]. In situ simulations can identify multiple potential operational gaps, which allow decision-makers to take necessary action in time [1]. There are now significant pieces of evidence demonstrating that simulation education interventions can produce changes in provider knowledge, attitudes, and skills in both the simulation laboratory and clinical settings [3-8]. Not only does simulation training provide learning at the individual level, but it also has an integral part to play in systems testing. Every session holds the potential to learn and improve on the systems level, and simulation can be a useful tool in the development of new standard operating procedures and policies needed to respond to a crisis [9].

During previous epidemics, such as Ebola, simulation programs had a major role in health system readiness and contributed effectively to the establishment of critical strategic and preparedness plans. These plans included the rapid development and utilization of a comprehensive simulation-based training program for healthcare professionals (HCPs) [10].

Second to identifying the need for training, simulation is a powerful tool in managing the COVID-19 crisis, as it can not only ensure patient safety but also provide a safe learning and training environment for HCPs to develop practical skills to deal with COVID-19. Simulation and online learning played a major role in dealing with COVID-19 in its early stages in China [11].

The main objective of our program was to assess the hospital operational readiness by conducting a series of in situ simulations in different areas of our hospital that deal with pediatric COVID-19 patients. In this report, we describe implementation steps, challenges, and solutions for simulation programs for HCPs to make a difference in the response to COVID-19 at a pediatric tertiary hospital in Saudi Arabia and share ways in which a simulation program can be utilized in healthcare organizations operating under pressure.

## Materials and methods

Running simulations to improve preparedness for COVID-19

King Abdullah Specialized Children’s Hospital (KASCH) is the largest pediatric tertiary-care medical center under the Ministry of National Guard - Health Affairs (MNG-HA) in Saudi Arabia. Pediatric Simulation Training and Education Program (PediSTEP) in KASCH is a multidisciplinary simulation program that includes nursing services, different specialties of physicians, as well as other services, such as emergency services, respiratory therapy, and an infection control department. It offers different types of simulation programs such as high-fidelity and low-fidelity simulation, interprofessional simulation, procedural sessions, quality simulation-based programs, and in situ simulation.

Based on our program’s endeavor to provide high-quality and efficient training and education for HCPs, we believe simulation can play a major role in readiness for the global COVID-19 crisis and any potential similar future pandemics.

COVID-19 has been spreading rapidly across the world and pediatric cases are increasing in our hospital, making immediate training and preparedness testing of utmost importance.

To assess hospital operational readiness, we conducted a series of in situ simulations in different areas of the hospital that dealt with pediatric COVID-19 patients, such as pediatric emergency room, operation room, pediatric intensive care unit, and pediatric wards. We involved in these sessions all HCPs who usually care and deal with pediatric patients, including physicians, nursing staff, respiratory therapists, anesthesia technicians, and radiology technicians.

Clinical, quality improvement, and infection control departments were engaged in this simulation program. The clinical departments that were directly involved with the effort included personnel from emergency medicine, pediatric critical care, operating room, and pediatric wards, as well as nursing services.

There were two major goals for this COVID-19 simulation program. First, simulation can play a role in understanding and testing the robustness of our systems such as infection control measures and flow of patients from emergency room to pediatric wards or pediatric intensive care unit, and optimizing workflows, dependencies, etc. (system focus). Second, simulation can help HCPs in safely and effectively handling patients with COVID-19, and in supporting HCPs in dealing with the emotional strain of the situation (personal focus).

Aims of our COVID-19 simulation program:

- To offer a simulation for HCPs to test and prepare them for a possible escalation of the COVID-19 situation.

- To prepare and reassure staff that the management of COVID-19 cases can be carried out safely.

- To reduce the cognitive load of HCPs as the workload escalates and to ensure a coordinated response.

To accomplish these goals, a COVID-19 simulation-based program was developed and run by PediSTEP team facilitators, including physicians, nurses, and infection control practitioners. We designed seven scenarios to include different clinical situations of pediatric COVID-19 cases, such as emergency room triage and respiratory support (Table 1).

**Table 1:** COVID-19 simulation-based scenarios

Scenario title	Environment	Target group
Aerostation/noninvasive ventilation	Pediatric ward	Nurses, physicians, respiratory therapists
Intubation	Pediatric intensive care unit, emergency room	Physicians, nurses, respiratory therapists, radiology technicians
Intubation/extubation	Operation room	Physicians, nurses, anesthesia technicians
Mechanical ventilation care	Pediatric intensive care unit	Physicians, nurses, respiratory therapists
Transportation	From pediatric ward to pediatric intensive care unit	Physicians, nurses, respiratory therapists
Cardiopulmonary resuscitation	Emergency room, pediatric intensive care unit	Physicians, nurses, respiratory therapists
Triage	Emergency room	Nurses

An evaluation template was formed by the PediSTEP team to unify the qualitative evaluation step. This evaluation form includes many items related to the team performance issue, infection control measures, and system issues. It rated the items as done, partially done, and not done, with a comments box for further notes. These simulations have been conducted by an expert team from PediSTEP members.

The simulations occurred during a normal working day and were run using a full-body manikin and the real settings and resources of the involved areas, which included the emergency department, pediatric wards, pediatric intensive care unit, and the operation room. These in situ simulations allowed us to include regular HCPs working that day within their real environment. The participating HCPs included physicians, nursing staff, respiratory therapists, anesthesia technicians, and radiology technicians.

After the simulation was completed, the participants were debriefed by PediSTEP facilitators.

## Results

In total, seven simulation-based drills were conducted during March 1-30, 2020, and 89 HCPs participated in these sessions (Table 2).

**Table 2:** Characteristics of healthcare providers who participated in COVID-19 simulation-based scenarios

Participants	N (%)
Total	89 (100)
Physicians	17 (19)
Nursing staff	56 (63)
Respiratory therapists	10 (11)
Radiology technicians	3 (3)
Anesthesia technicians	3 (3)

These series of multidisciplinary in situ simulations exposed many issues that were qualitatively assessed and presented to related departments and administrators, so action plans were created. Many of the latent threats revealed by our simulations were regarding teamwork, workflows, and adherence to infection control measures, such as control of crowdedness inside the patient’s room, proper donning and doffing of the personal protective equipment (PPE), equipment availability (e.g., a high-efficiency particulate air filter), and communication among the HCPs.

These sessions received encouraging feedback through the PediSTEP electronic feedback form, which was distributed after each session. Most trainees revealed in their comments that the simulation exercises enhanced confidence and reduced anxiety among the pediatric HCPs during the COVID-19 crisis. Even while witnessing the impact of the virus on adult patients, the trainees reported that the exercises helped to normalize the processes required to care for pediatric patients with COVID-19 and afforded them an opportunity to become familiar with the organization’s new policies.

## Discussion

Challenges and solutions

We were planning to continue to use in situ simulation as the COVID-19 outbreak progresses, but this simulation is resource-consuming, in time, human resources, and medical supplies, especially during the current acute phase of a disease outbreak.

Our major challenge was the large number of HCPs that require training and the limited training resources of PPE, for which demand exceeds availability in most current healthcare systems, and these resources should be preserved for the real situations of the COVID-19 crisis, not consuming them in simulation training [12].

As these simulations were performed on the clinical unit during work hours, another issue raised is the need to balance training needs with the current work status of the participants. Therefore, to match needs, challenges, and methods to implement effective interventions, a novel way of enacting simulations was established. Thus, instead of in situ simulation sessions, we initiated video-recorded modules for each of these scenarios, which became available online through the PediSTEP webpage in the MNG-HA home page as E-learning modules for all KASCH staff (approximately 1,500 HCPs).

A spectrum of educational simulation-based online resources included different COVID-19 scenarios that were used to educate and verify both institutional and provider readiness in the identification and care of patients suspected of or diagnosed with COVID-19. This allowed us to ensure the appropriate use of resources, maintain homogeneous education, and enable effective patient care. This gave healthcare workers the opportunity to watch it in their free time or through their working hours and allow for self-paced learning.

There were several outcomes of this simulation program. First, it allowed us to identify potential pitfalls and challenges in the management of COVID-19 patients during their stay at our hospital. Second, the simulation program helped to increase the awareness and exploration of existing hospital guidelines and in developing new guidelines related to COVID-19. Third, it helped to improve the readiness of HCPs in crisis situations and in preparing them to protect themselves and save valuable resources.

The final program currently uses blended learning solutions to optimize efficiency and effectiveness. The initial vision was carried through the final design and consisted of requisite parts that included online web-based content and in situ training of simulation activities. We found that in situ simulations identified multiple latent hazard issues, and it allowed us to take corrective action before the COVID-19 crisis developed.

## Conclusions

Simulation has great potential to positively impact hospital preparedness, training and educating HCPs in the pediatric field, and mitigating the negative effects of the COVID-19 crisis and for any future crisis situations. The video-recorded simulations method is a good alternative to in situ simulations to maintain medical supplies during the COVID-19 crisis.

We believe that this report may serve as an example for other simulation programs that are truly integrated with healthcare delivery systems and that can engage in significant, collaborative training development activities in response to operational needs.
